# European Correspondence

**Published:** 1878-09

**Authors:** R. J. Nunn

**Affiliations:** Paris, France


R. J. Nunn.

---

Paris, France, Aug. 3d, 1878.

The microscope is to-day an absolute necessity to the accomplished physician ; cases may occur at any moment in which it may be his only means of diagnosis, and without it he will be only ignorantly stumbling in the dark. It is very far from my desire to convey the idea that each physician should be a skilled microscopist. That is impossible. It would be absurd to expect it. Ridiculous in the busy practitioner to attempt it, because the study of any one department of microscopy has now become wide enough to engage the entire attention of one person, but what I do consider possible and necessary is that each physician shall be acquainted with the manner of mounting microscopic specimens which can be sent to specialists at a distance and enable him to bring to bear upon his case the experience of the world, if necessary. This is practicable, possible, and useful, and if to this is added a fair knowledge of minute pathology, it is all that can be required. A curious case or two occurring in the practice of some of the most celebrated Eurepeon practitioners which have come to my knowledge and of which I have seen specimens, will serve to illustrate my meaning.

Here is a case of pus in the urine—from whence comes it? The quantity is but small to be sure, but then it is, and a dozen examinations fail to detect any of the epitheliums of the urinary organs; then came the idea that perhaps it might be one of the more delicate epithelia which would be destroyed by laying in urine; drops of urine were obtained fresh as secreted by the patient; the preparations were so mounted as to fix the elements present and the microscope then demonstrated the presence of epithelium of the pancreas, in short, carcinoma of the pancrea opening into urinary apparatus was diagnosed, of course an unfavorable prognosis was given and it is needless to say the patient died.

Another case having all the appearance, and supposed to be pus in the urine, proved upon careful scientific examination to be the white globules of the blood.

A most curious case was one in which a patient was attacked with swelling of one leg, apparently cutaneous, then vesicles appeared, which, when examined by transmitted light, seemed to have one or two little papilla running into each, when punctured a drop of serum exuded, which if wiped away was succeeded by another and this could be repeated apparently indefinitely. If left to themselves the vesicles either burst and then dried, forming a crust, or else dried and formed a crust without bursting. I need not say that the attendant was "non plussed" and would not say what the case was, but the microscope showed white blood globules in the serum of the vesicles, the case was diagnosed as a disease of the lymphatics, treated and cured.

In the practice of M. Vaisin, at Salpetriere, I saw the injection of sheep's blood (venvus) into the cellular tissue used as a substitute for transfusion. This is the fourth case in which M. Vaisin has employed this remedy, one of which was unsuccessful, the patient being in "articulo mortis." It is to be hoped this may prove a sufficient substitute for transmission over which it has the great recommendation of extreme simplicity, nothing being necessary but to fill a syringe with the warm venous blood and inject it through a canula into the tissue, covering the aperture with a bit of plaster. Into the case I saw, about one and a half or two ounces were injected, and the sole precaution required is to avoid the use of arteriel blood, a very small quantity of which, M. Voisin assured me, would result in the formation of an abscess.

Speaking of transfusion, I saw the other day a most melancholy case. Going into the rooms of a medical friend, I met a young man, who, without having the build or figure of a consumptive, had all the other appearances of the disease most unmistakably. After he left I enquired his history and was told that he was a physician, who previously healthy, and without the slightest taint in his family of hereditary disease, had allowed transfusion to be performed for the benefit of a wounded man, a patient in one of the hospitals. He was shortly afterwards attacked with tubercles in both lungs, and is now himself just fighting with death.

Here is something decidedly new in therapeutics. I translate from a morning journal: "A few days since we described a substance sold as a plaything for children at two cents, which had the property of enflaming when coming in contact with any liquid."

"A singular communication has been made to us on this subject as follows:

"It appears that in Germany this substance is employed to correct children who have the deplorable habit of forgetting themselves in bed.

"When a child is subject to incontinence, they place under the sheet a very small lot of the substance in question. At the first contact with liquid the substance enflames, and the child, slightly burnt, is reminded of his imprudence, and corrects it. This means is sharp, but very efficacious, say the Germans."

The application of the remedy, if not exactly practicable, has at least the merit of novelty, originality and ingenuity.

At the clinic of M. Onimus, I saw several interesting cases under electrical treatment. One in particular, a case of general glandular enlargement of the right side, involving all the glands from the parotid to the axilla, was being treated by electrolysis, the result so far being favorable. M. Onimus employs for the constant current a very compact form of the Daniel element modified by himself.

Here I would like to mention that for some years, certainly five or six, I have altogether discarded the use of needles in electrolysis. I found them difficult to keep in order. If made of steel, they rusted, in spite of gilding, etc.; if of gold they were too soft to penetrate the skin, and finally the tissues clung to the non-conducting varnish with which they were coated (except the points) in a most inconvenient way. To remedy these evils I employ a fine trocar and canula, or a hollow needle, and a wire coated with varnish except about half an inch at each end. The trocar and canula being introduced, the trocar is withdrawn, the canula being left in position. Through the canula the coated wire is now introduced, and held in place while the canula is withdrawn, and finally the connection is established with the outer end of the wire by a small binding screw, or otherwise. For coating the wire, I have found collodion very convenient, and it answers every purpose. This method has another great advantage, *i. e.*, that any metal, however soft, may be used for the wire, and so zinc may be deposited in the center of a tumor by using a zinc wire, which is not unfrequently a desirable plan of treatment.

In the gynæcological clinic of M. Cheron, some interesting cases were presented. Here I found the duck-bill speculum in use, and galvanism is much in requisition. The battery employed is peculiar to M. Cheron, and consists of a piece of carbon in a porous cell, into which is packed binoxide of manganese and protochloride of mercury. Water and zinc in the outer jar complete the combination, which M. Cheron says only acts when the circuit is closed, and will last for two years. In this practice the Hodge pessary appears to be the favorite, at least for retro and antiversions. Mennorrhagia is successfully treated by the interrupted continuous current. Epithelial inflamations of the os and cervix are combatted with alcoholic solution of prussic acid or with salicylic acid, and some interesting cases of vaginitis and blennorrhagia, as the result of spinal irritation, were shown, which, though not modified by local treatment, were finally subdued by treating the spinal irritation. The medication adopted here for piles is the internal administration of capsicum and the local application of chalk ointment and douches.

R. J. NUNN.

---

TREATMENT OF DALTONISM BY FUCHSINE.—M. Delbœuf has found that when a person afflicted with color-blindness looks through a solution of fuchsine, his infirmity disappears. M. Javal has turned this discovery to advantage by interposing a thin layer of gelatine colored with fuchsine between two glasses. It is claimed that the daltonic infirmity is corrected by the use of these glasses.—*La France Medicale.*

